# Selective Inhibition of PI3K/Akt/mTOR Signaling Pathway Regulates Autophagy of Macrophage and Vulnerability of Atherosclerotic Plaque

**DOI:** 10.1371/journal.pone.0090563

**Published:** 2014-03-05

**Authors:** Chungang Zhai, Jing Cheng, Haroon Mujahid, Hefeng Wang, Jing Kong, Yue Yin, Jifu Li, Yun Zhang, Xiaoping Ji, Wenqiang Chen

**Affiliations:** The Key Laboratory of Cardiovascular Remodeling and Function Research, Chinese Ministry of Education and Chinese Ministry of Health, Shandong University Qilu Hospital, Jinan, Shandong, China; Chang Gung University, Taiwan

## Abstract

Macrophage infiltration contributes to the instability of atherosclerotic plaques. In the present study, we investigated whether selective inhibition of PI3K/Akt/mTOR signaling pathway can enhance the stability of atherosclerotic plaques by activation of macrophage autophagy. In vitro study, selective inhibitors or siRNA of PI3K/Akt/mTOR pathways were used to treat the rabbit's peritoneal primary macrophage cells. Inflammation related cytokines secreted by macrophages were measured. Ultrastructure changes of macrophages were examined by transmission electron microscope. mRNA or protein expression levels of autophagy related gene Beclin 1, protein 1 light chain 3 II dots (LC3-II) or Atg5-Atg12 conjugation were assayed by quantitative RT-PCR or Western blot. In vivo study, vulnerable plaque models were established in 40 New Zealand White rabbits and then drugs or siRNA were given for 8 weeks to inhibit the PI3K/Akt/mTOR signaling pathway. Intravascular ultrasound (IVUS) was performed to observe the plaque imaging. The ultrastructure of the abdominal aortic atherosclerosis lesions were analyzed with histopathology. RT-PCR or Western blot methods were used to measure the expression levels of corresponding autophagy related molecules. We found that macrophage autophagy was induced in the presence of Akt inhibitor, mTOR inhibitor and mTOR-siRNA in vitro study, while PI3K inhibitor had the opposite role. In vivo study, we found that macrophage autophagy increased significantly and the rabbits had lower plaque rupture incidence, lower plaque burden and decreased vulnerability index in the inhibitors or siRNA treated groups. We made a conclusion that selective inhibition of the Akt/mTOR signal pathway can reduce macrophages and stabilize the vulnerable atherosclerotic plaques by promoting macrophage autophagy.

## Introduction

Atherosclerotic plaque rupture is the major cause of acute cardiovascular events, which is characterized by a thin fibrosis cap (<65 µm) and a large necrosis core with plenty of macrophages and T lymphocytes invasion, so the treatment goal in stabilizing vulnerable plaques is of great clinical importance [1]. Therefore, establishment of strategies aimed at thickening fibrosis cap or eliminating intrusion cells in the lipid core is crucial. In the development of atherosclerotic plaques, macrophage could drive lesion progression, destabilization, and rupture by producing and releasing various cytokines and growth factors such as matrix metalloproteinase (MMPs), tumor necrosis factor (TNF-α) and Interferon-γ (IFN-γ) [2]. In this way, treatment aimed at clearance of macrophages without influencing the fibrosis cap is very meaningful. Systemic therapy with statins has been shown to reduce but do not eliminate macrophages from atherosclerotic plaques [3]. Verheye et al found that stent-based delivery of everolimus, an inhibitor of mammalian target of rapamycin (mTOR), selectively cleared macrophages in rabbit atherosclerotic plaques by autophagy [4].

Autophagy, which is an evolutionary conserved process involved in the degradation of long-lived proteins and excess or dysfunctional organelles, is a kind of cell death different from apoptosis and necrosis. However, despite of the growing interest in autophagy, its role in atherosclerosis still remains poorly understood [5]. Most likely, autophagy protects plaque cells against cellular distress, especially oxidative injury by degrading the damaged intracellular components. Defect in autophagy also induces enhanced inflammation particularly in those with high blood cholesterol [6]. Because atherosclerosis is an inflammatory disorder of the arterial intima and initiated by high cholesterol, therefore the normal function of autophagy is important for homeostasis. The regulation of autophagy is complicated and there are several pathways that are linked to it. Phosphoinositide 3-kinase/protein kinase B/mammalian target of rapamycin (PI3K/Akt/mTOR) pathway is closely related in regulation of autophagy for its role in cell survival, proliferation and differentiation. Much work has been done on this pathway but the exact role in atherosclerosis still remains unclear [7].

In the present study, we investigated whether selective inhibition of PI3K/Akt/mTOR signaling pathway can inhibit the atherosclerosis progression and enhance the stability of atherosclerotic plaques by activation of macrophage autophagy.

## Results

### Vitro experiments

To prove our hypothesis that selective inhibition of PI3K/Akt/mTOR signaling pathway can facilitate macrophage autophagy, rabbit's peritoneal primary macrophage cells were cultured and rabbits were used in our vivo experiment. We used selective drugs of PI3K inhibitor LY294002, Akt inhibitor triciribine (API-2), mTOR inhibitor rapamycin and mTOR-siRNA to promote autophagy of macrophages.

#### Macrophage autophagy was induced in the presence of API-2 (group B1), rapamycin (group C1) and mTOR-siRNA (group D1) respectively while inhibited by the effect of LY294002 (group A1)

**Figure 1:**
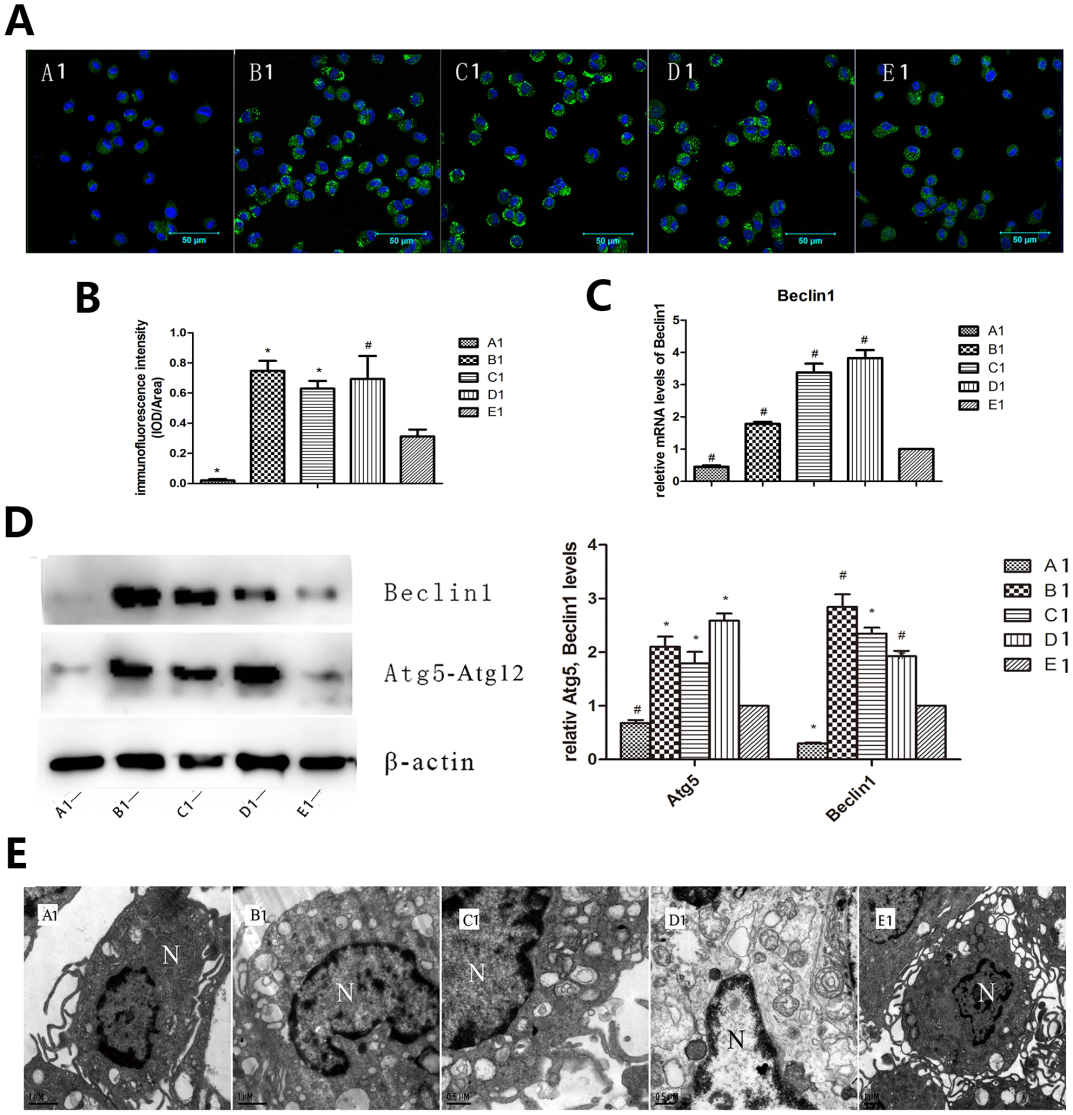
Autophagy was induced in the presence of API-2, rapamycin and mTOR-siRNA respectively and inhibited in response to LY294002. Rabbit peritoneal macrophages were obtained to verify if blockage of PI3K, Akt, mTOR molecules respectively could promote autophagy. (A, B) Cell immunofluorescence staining of LC3-II among different groups (×400) showed that increased LC3-II punctate dots appeared in B1, C1 and D1 groups while decreased punctate dots in group A1 compared to group E1. (C) Expression of autophagy related gene Beclin 1 detected by QRT PCR showed remarkable higher Beclin 1 mRNA in group B1, C1 and D1 and lower in group A1 than group E1. (D) Western blot showed that expression of autophagy protein Beclin 1 and Atg5-Atg12 conjugation increased in group B1, C1 and D1 and decreased in group A1 than group E1. (E) Transmission electron microscope showed an intact nonpyknotic nucleus and numerous vacuoles, large cytoplasmic inclusions and myeline figure in the cytoplasm in group B1, C1 and D1, whereas group A1 and E1 did not display vacuolization but a little was observed (base level autophagy). Endoplasmic reticulum and mitochondria displayed varying degrees of swelling in group B1, C1 and D1. The “N” represents the nuclear. A1: LY294002; B1: triciribine; C1: rapamycin; D1: mTOR-siRNA; E1: control. ^*^ *P*<0.01 vs control, ^#^ *P*<0.05 vs control.

Cell immunofluorescence staining was used to see protein 1 light chain 3 II dots (LC3-II). The detection of LC3-II is usually used as a mark of autophagy activation because it is a structural protein vital in autophagosome formation. Compared to the control group, group A1 showed significantly decreased LC3-II punctate dots, on the contrary, there were increased number of dots seen in group B1, C1 and D1 (*P*<0.05∼0.01, Fig. 1A, 1B). Besides LC3-II, Beclin 1 was analyzed as sign of autophagy both in the participant and regulation of autophagosome. Atg5-Atg12 conjugation was detected because the binding of LC3 to the phagophore membrane is dependent in the presence of an Atg12-Atg5-Atg16 complex. We found that the mRNA expression of Beclin 1 increased significantly in the group B1, C1, and D1 and decreased in group A1 as compared to the control group (*P*<0.05, Fig.1C). Moreover, Western blot results showed the protein expression levels of Beclin 1 and Atg5-Atg12 conjugation were remarkably lower in group A1 and much higher in group B1, C1 and D1 than that of the control group (*P*<0.05∼0.01, Fig. 1D). Transmission electron microscope was used to see direct sign of autophagy, which is usually known as a gold standard for autophagy formation. Triciribine, rapamycin and mTOR-siRNA treated macrophages showed an intact nonpyknotic nucleus and numerous vacuoles, large cytoplasmic inclusions and myeline figure in the cytoplasm which are characteristics of autophagy, whereas LY294002 treated and control macrophages did not display vacuolization but a little was observed (base level autophagy). Endoplasmic reticulum and mitochondria displayed varying degrees of swelling in group treated with triciribine and rapamycin and mTOR-siRNA (Fig. 1E).

#### Cytokines secreted by macrophages and effect of different inhibitors on Akt and mTOR molecules

**Figure 2:**
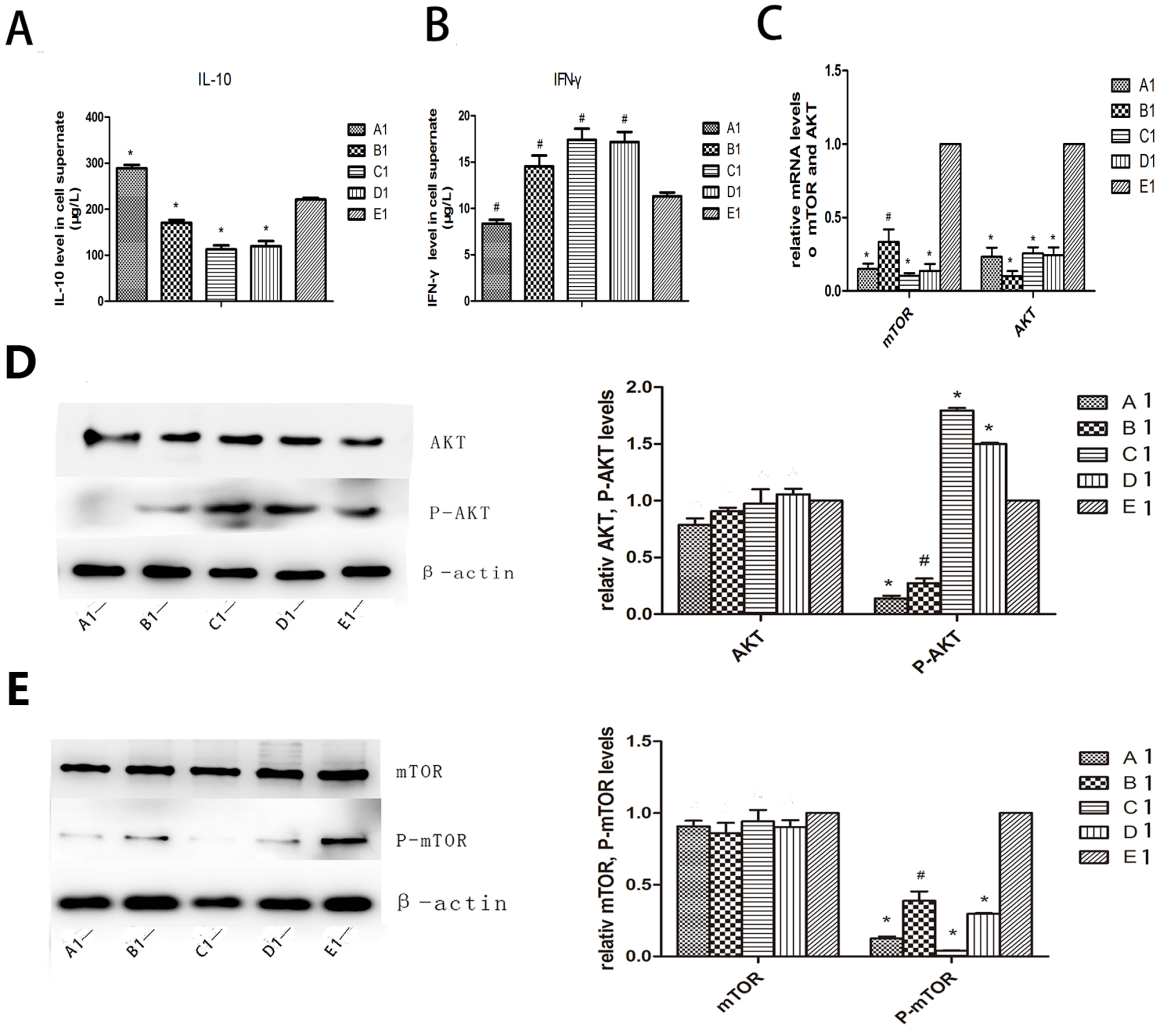
Cytokines secreted by macrophages and effect of different inhibitors on Akt and mTOR molecules. (A, B) ELISA assay of cytokines in rabbit primary macrophage supernate showed an increased level of IL-10 and decreased level of IFN-γ in group A1 than E1. On the contrary, level of IL-10 was lower in group B1, C1 and D1 compared to group E1 whereas IFN-γ was higher. (C) QRT PCR showed that mRNA level declined in each group compared to group E1. (D, E) Western blot revealed that total Akt and total mTOR did not change among the five groups while phosphorylation of the Akt and mTOR reduced significantly in the experimental groups compared to the control. However, Akt in group C1 and D1 were hyperphosphorylated. A1: LY294002; B1: triciribine; C1: rapamycin; D1: mTOR-siRNA; E1: control. ^*^ *P*<0.01 vs control, ^#^ *P*<0.05 vs control.

We used enzyme linked immunosorbent assay (ELISA) to detect levels of IL-10 and IFN-γ secreted by macrophages after co-culture with drugs and mTOR-siRNA. IL-10 plays a role in inhibiting starvation-induced autophagy while IFN-γ has an opposite effect. In our experiment, IL-10 increased in group A1, and declined evidently in group B1, C1 and D1 compared to the control group; IFN-γ level decreased in group A1 and increased in group B1, C1 and D1 compared to group E1 (*P*<0.05∼0.01, Fig. 2A, 2B). We also observed Akt and mTOR with quantitative reverse-transcription polymerase chain reaction (QRT-PCR) and western blot. The mRNA expression levels of Akt and mTOR were significantly lower in group A1, B1, C1 and D1 than that of the control group (*P*<0.05∼0.01, Fig.2C). Western blot results showed that the total Akt and mTOR did not change among the five groups while phosphorylation of the Akt and mTOR reduced significantly in the experimental groups as compared to the control group (*P*<0.05∼0.01, Fig. 2D, 2E). Apparently Akt in group C1 and D1 were hyperphosphorylated (*P*<0.01, Fig. 2D).

### Vivo experiments

#### There was no statistical difference of blood lipid profile found in rabbits in our experiments and in the control group but the incidence of plaque rupture was significantly less in the treatment groups

**Figure 3:**
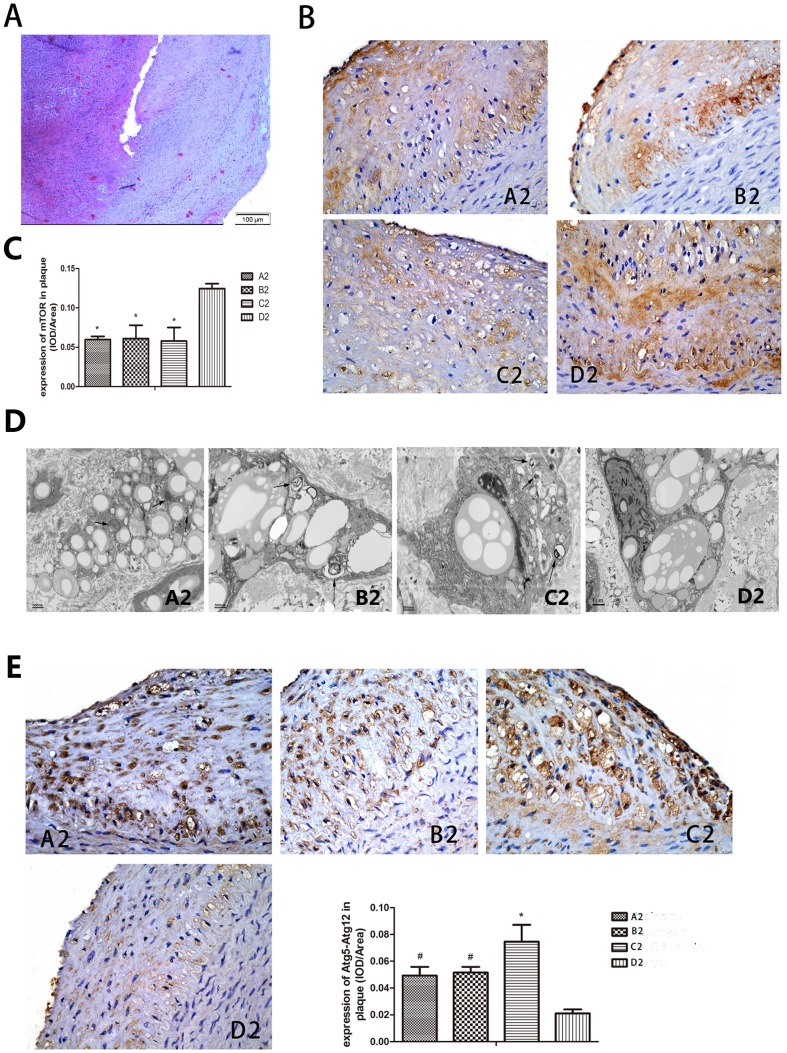
Effect of Pharmacological triggers in the experiment showed the rupture of plaque incidence higher in control group. IH staining and transmission electron microscope showed mTOR decreased and autophagy increased in experimental group. Pharmacological triggers were done by 0.15·kg^−1^ of Chinese Russell's viper venom injecting intraperitoneally, 30 min later, 0.02 mg·kg^−1^ histamine was injected intravenously. (A) Haematoxylin and eosin staining of the cross section of the abdominal aorta in a rabbit of group D2 showing plaque rupture and thrombosis. (B, C) IH staining showed mTOR decreased obviously in group A2, B2 and C2 than D2. (D) Transmission electron microscope analysis showed macrophage autophagy was enhanced in group A2, B2 and C2 compared to group D2. Arrows in picture B2, C2 and d2 represent vacuoles with cytoplasmic inclusions and myeline figure. The “N” represents the nuclear. (E) IH analysis of Atg5-Atg12 conjugation found percentage of positive-stain cells was significantly increased in group A2, B2 and C2 in comparison to group D2. A2: triciribine; B2: rapamycin; C2: mTOR-siRNA; D2: control; ^*^ *P*<0.01 vs control, ^#^ *P*<0.05 vs control.

Four rabbits [1 in Akt inhibitor triciribine group (group A2), 1 in mTOR inhibitor rapamycin group (group B2), and 2 in mTOR-siRNA group (group C2) and 0 in the control group (group D2), respectively] died accidently from anesthesia or by operation during the experiment. The weight of rabbits was 2.21±0.15 kg at baseline but increased to 3.55±0.21 kg at week 16 (*P*<0.05). However, there was no significant difference in body weights among the four groups at either baseline or week 16. The blood lipid profile was not statistically significant among the four groups after drug treatment or gene intervention (Table 1). Plaque rupture was defined as fibrous cap disruption with luminal thrombosis or a buried fibrous cap within a plaque [8]. In the end of the experiment, after pharmacological triggering, no rabbit in group A2, 1 in group B2 and 1 in group C2 (1/9, 11%; 1/8, 13%), but 6 in group D2 (6/10, 60%) suffered abdominal aortic plaque rupture (all *P*<0.05, Fig. 3A).

**Table 1:** Table Serum lipid Profiles of the rabbits in the four groups at week 16.

Parameters	GroupA2 (n = 9)	GroupB2 (n = 9)	GroupC2 (n = 8)	GroupD2 (n = 10)
TC(mmol/l)	26.60±8.42	29.08±7.14	28.76±5.24	29.12±6.80
TG(mmol/l)	2.94±1.71	3.11±1.60	2.77±1.91	3.19±1.75
HDL(mmol/l)	1.75±1.12	1.98±1.03	1.94±1.25	1.84±0.71
LDL(mmol/l)	24.82±7.33	27.21±4.56	25.7±3.21	27.74±3.54

Group A2 is Akt inhibitor triciribine treatment group, Group B2 is mTOR inhibitor rapamycin treatment group, Group C2 is mTOR-siRNA group and Group D2 is the control group.

TC, total cholesterol; TG, triglyceride; HDL, high density lipoprotein; LDL, low density lipoprotein.

#### Detection of mTOR and autophagy related genes after rabbits were fed on drugs or injected with mTOR-siRNA

Immunohistochemistry (IH) staining showed an obvious decrease of mTOR in group A2, B2 and C2 than D2, which clearly showed an inhibition of the pathway (all *P*<0.01, Fig. 3B, 3C). Transmission electron microscope analysis together with IH analysis was made to confirm the activation of autophagy. Similar results were obtained as vitro results under transmission electron microscope: vacuoles with cytoplasmic inclusions and myeline figure displayed in the atherosclerosis segment after rabbits were treated with triciribine, rapamycin and mTOR-siRNA (Fig. 3D). IH analysis found percentage of positive-stain cells for Atg5-Atg12 conjugation was significantly increased in group A2, B2 and C2 as comparison to group D2 (*P*<0.05∼0.01, Fig. 3E). The results indicated an increased autophagy occurred in the three treated groups.

#### Macrophage autophagy promoted vulnerable plaque stability via lower plaque burden, smaller vulnerability index and alleviated inflammatory response

**Figure 4:**
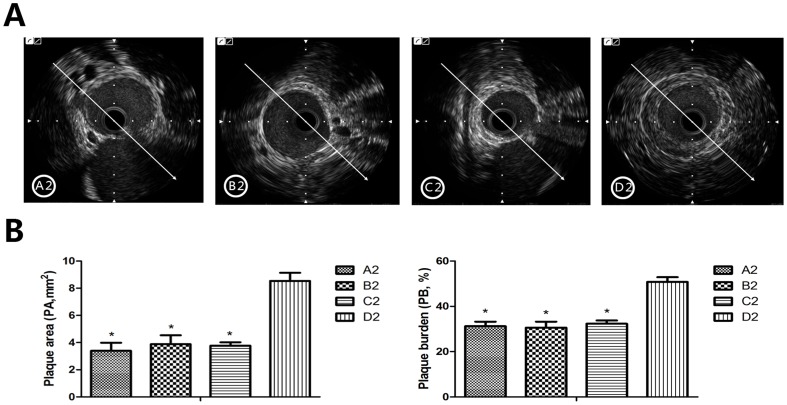
Macrophage autophagy promotes vulnerable plaque stability via decreased plaque burden and macrophages. IVUS measurement was taken to measure lumen area (LA), external elastic membrane area (EEMA), plaque area (PA) and plaque burden (PB) (PA =  EEMA – LA, PB% =  PA/EEMA ×100%). (A) IVUS image showed that the plaque area was huge in group D2 than that of A2, B2 and C2. (B) PA and PB calculated by LA and EEMA. We can see PA in the abdominal aorta in group A2, B2 and C2 was significantly lower than that in group D2, and PB% was remarkably reduced in group A2, B2 and C2 compared to group D2. However, the three groups did not differ in PB%. A2: triciribine; B2: rapamycin; C2: mTOR-siRNA; D2: control; ^*^ *P*<0.01 vs control.

Intravascular ultrasound (IVUS) was used to measure plaque burden (PB) which can be estimated by lumen area (LA), external elastic membrane area (EEMA) and plaque area (PA) (PA = EEMA–LA, PB% = PA/EEMA×100%). Our previous study showed that PA measured by IVUS was a predictor of plaque rupture [9]. Measurements in the present study showed that values of PA and PB% of the plaques in group A2, B2 and C2 were significantly lower than that in group D2, which indicated that Akt and mTOR inhibitors or mTOR-siRNA decreased the plaque vulnerability (all *P*<0.01, Fig. 4A, 4B).

**Figure 5:**
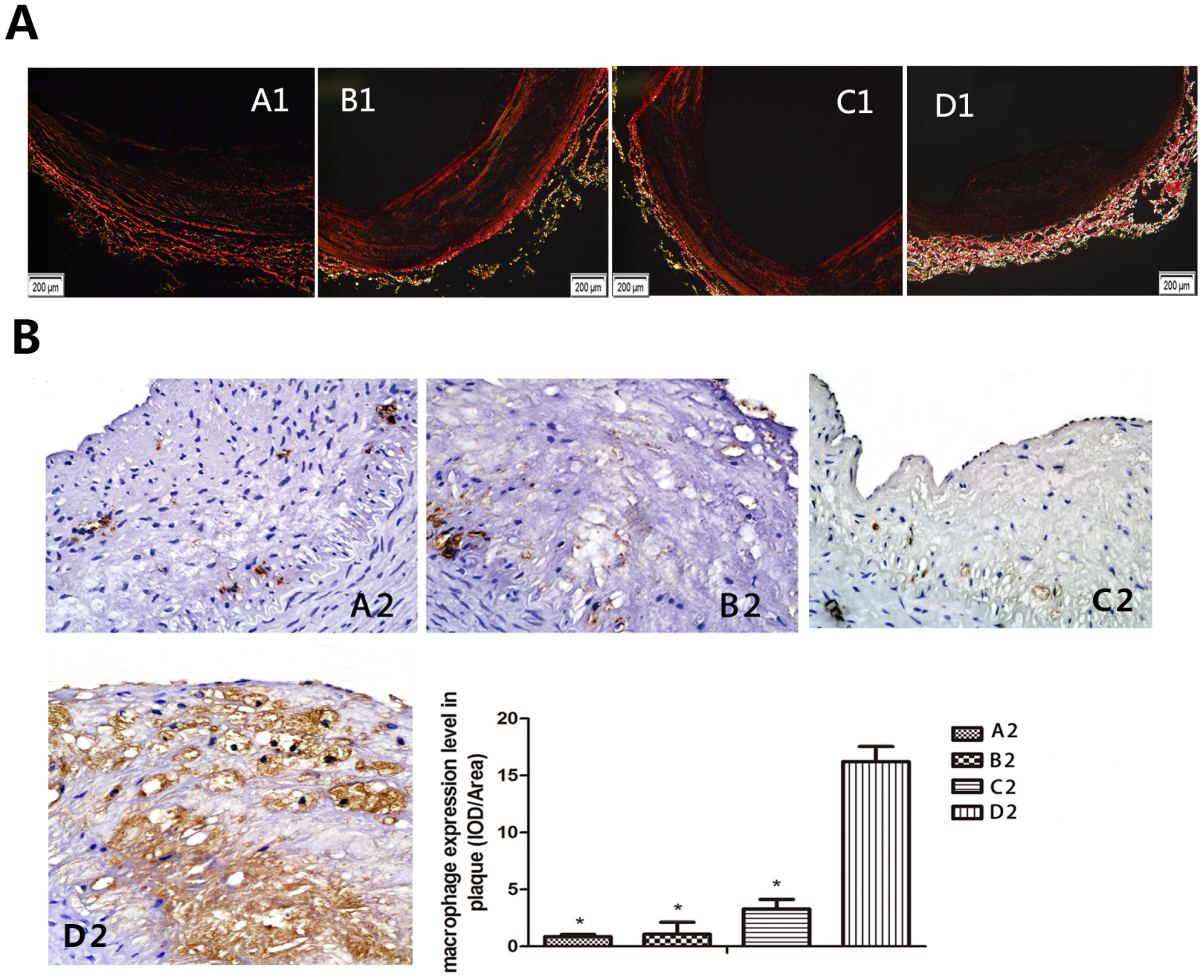
Sirius red staining for collagen and IH for macrophages in plaque. (A) The interstitial collagen content of aorta abdominals plaques was evaluated by Sirius red staining. Collagens are identified by birefringence under polarized light illumination following standard sirius red procedure. (B) IH for macrophage in plaque detected increased macrophages expressed in control group and less in group A2, B2 and C2. A2: triciribine; B2: rapamycin; C2: mTOR-siRNA; D2: control; ^*^ *P*<0.01 vs control.

**Figure 6:**
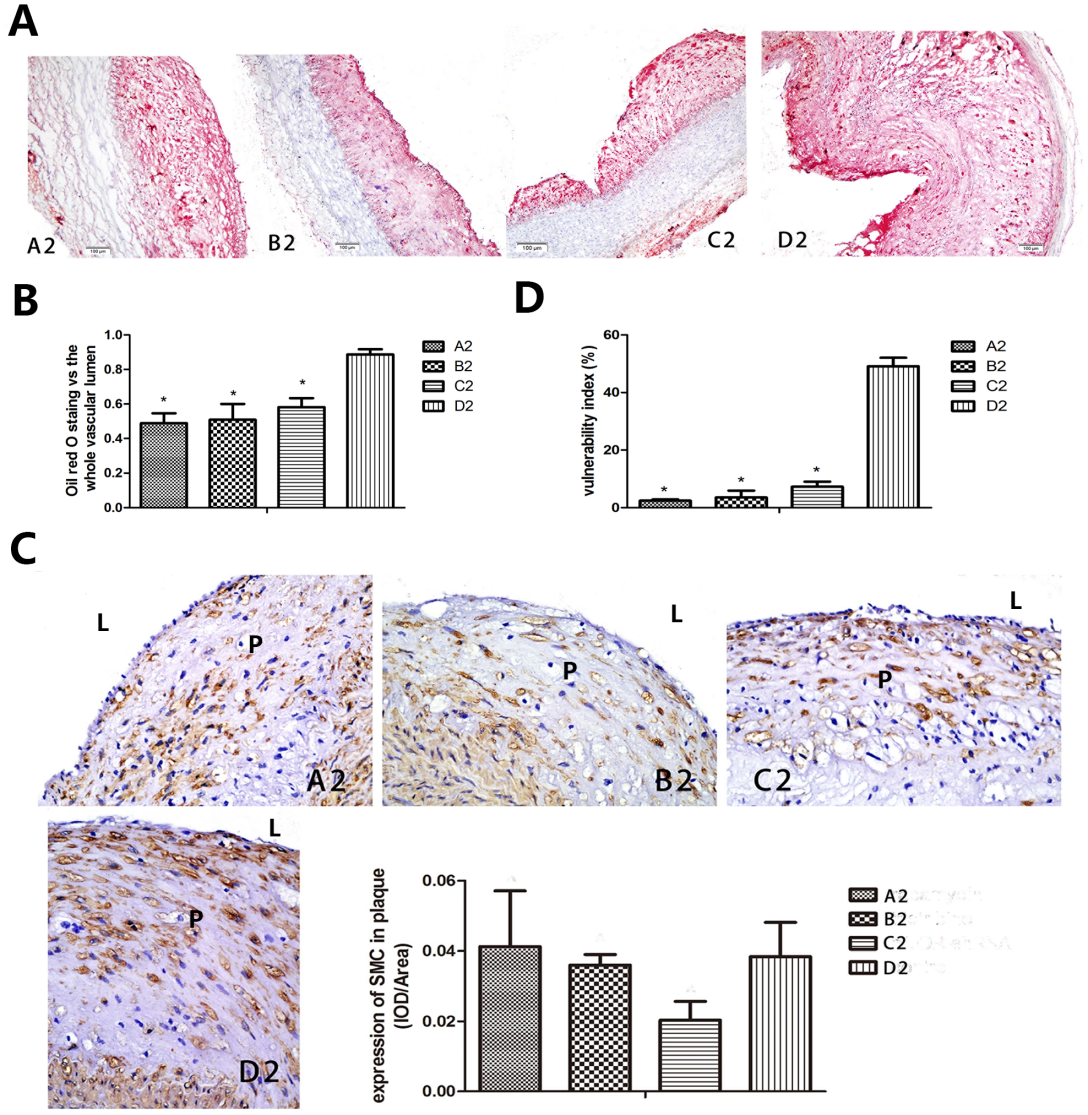
Macrophage autophagy induced by drugs and mTOR-siRNA alleviated vulnerability index. The vulnerability index can be calculated as: (macrophage staining %+ lipid staining %)/(smooth muscle cell %+ collagen fiber %). So we conducted Sirius red (Fig. 5) and oil red O staining together with IH for macrophages (RAM-11) (Fig. 5) and smooth muscle cells (α-SMA). (A, B) Oil Red O Staining was taken for lipid content evaluation and was expressed as a percentage verse the whole vessel lumen. Our result revealed much smaller plaque in aorta abdominals in group A2, B2 and C2 compared to group D2. (C) IH analysis of α-SMA amount did not detect difference in plaque area among the four groups. L represents for vascular lumen, P represents for plaque. (D) Vulnerability was calculated by macrophage, lipid, α-SMA and collagen. It was significantly lower in the experimental group than the control group. A2: triciribine; B2: rapamycin; C2: mTOR-siRNA; D2: control; ^*^ *P*<0.01 vs control.

The vulnerability index (VI) of the plaque was also used as an important marker in predicting plaque rupture, which is calculated as: VI =  (macrophage staining %+ lipid staining %)/(smooth muscle cell %+ collagen fiber %) [10]. So we conducted sirius red and oil red O staining together with IH for macrophages (RAM-11) and smooth muscle cells (SMC). Sirius red staining of type I and type III collagen (Fig. 5A) and positive staining area of RAM-11 (all *P*<0.01, Fig. 5B) increased in the abdominal aortic segments in group A2, B2 and C2 than group D2, on the contrary, oil red O in the abdominal aortic segments decreased in group A2, B2 and C2 compared with group D2 (all *P*<0.01, Fig. 6A, 6B). The amount of SMC in plaque did not changed much in the treated group compared to the control group (Fig. 6C), as a result, the vulnerability index in group A2, B2 and C2 was significantly lower than that in group D2 (all *P*<0.01, Fig. 6D).

Cytokines such as MMPs secreted by macrophage can attenuate plaque stability and prompt rupture of atherosclerotic plaques. In the present study, we selected MMP9 instead, as it represents the other matrix metalloproteinase as well. IH experiment showed that the percentage of MMP9 positive-stained in the abdominal aortic sections was obviously lower in group A2 (7.27±2.95%), B2 (6.74±1.89%) and C2 (8.60±4.07%) than that in group D2 (25.35±12.06%) (all *P*<0.05), which meant an alleviated inflammation response caused by increased autophagy.

## Discussion

The major finding of the present study was that selective inhibition of PI3K/Akt/mTOR signaling pathway regulated macrophage autophagy and markedly affected atherosclerotic plaque inflammation, burden and vulnerability. Generally speaking, our study creatively inhibited the molecules of the pathway respectively both in vitro and in vivo. These molecular and cellular effects translated into a successful prevention of plaque disruption even in the presence of endothelial injury, hyperlipidemia and pharmacological triggering.

Studies have demonstrated atherosclerosis is a complex and perpetuating inflammatory disease and that plenty of macrophages invasion is an important feature of vulnerable plaques. Although statins show an anti-inflammation role in stabilizing vulnerable plaques, yet there is still not an optimal method available in vivo [11]. Therefore, treatment aimed in the clearance of macrophages in the atherosclerotic plaque without influencing the fibrosis cap is of great clinical importance.

Roles of autophagy in the process of atherosclerosis have drawn increasing attention to the researchers. Studies have found that in the early stage of AS, macrophage autophagy reduces the accumulation of foam cells thus inhibits plaque formation and development; moreover, in the middle and late stage of AS, macrophage autophagy reduces inflammation in the plaque and plays a role in plaque stabilization [12]. Verheye et al reported that mTOR inhibition by everolimus led to a selective clearance of macrophages within the atherosclerotic plaque without influencing the viability of SMC due to induction of macrophage autophagy [4].

Autophagy, the conserved pathway that maintains cellular quality control, plays a potential role in regulating lipoprotein metabolism such as obesity and atherosclerosis. Molecular mechanism study of autophagy has revealed that the regulatory molecules that control autophagy are varied, including ERK1/2, AMP kinase, class I and class III PI3K, Akt, mTOR and so on [13]. Phosphorylated class I PI3K enzymes and protein kinase B (Akt/PKB) can activate mTOR, and then its downstream effector p70S6 kinase as well as initiation factor 4E binding protein (4E-BP1) became activated. After 4E-BP1 was phosphorylated, it was separated from the eukaryotic initiation factor-4E (elF-4E). The latter is one part of the translation starting complex that promotes protein synthesis. Beclin 1 is a component of the class III PI3K complex that is required for autophagy. Study showed that Beclin 1 is functioned by anchoring on trans-Golgi network (TGN) [14]. It has been reported that mitogen-activated protein kinase c-Jun N-terminal kinase (JNK) might activate autophagy via Beclin 1 activity [15]. Extracellular signal-regulated kinase (ERK1/2) signaling also regulates autophagy and lysosome genes expression, and ERK8 has recently been demonstrated to stimulate autophagy by interacting with LC3 [16]. Detection of autophagy by electron microscope focuses on the mass vacuoles and myelin figures (concentric circle) formation. Other methods to detect autophagy include immunohistochemistry or molecular techniques such as analysis of GFP-LC3/Atg8 via fluorescence microscopy or lysates via western blot. Besides, presence of Atg5 which anchored to the phagophore membrane in an Atg12-Atg5-Atg16 complex and Beclin 1 which was essential in the formation of autophagosome were markers of autophagy [17].

Our study focused on the PI3K/Akt/mTOR pathway. We used inhibitors or mTOR gene silencing to selectively inhibit the molecules of the pathway both in vitro and in vivo. We discovered that macrophage autophagy was increased on the presence of Akt inhibitor, mTOR inhibitor or mTOR-siRNA in vitro study, while decreased after administrating PI3K inhibitor. In vivo study, we found that macrophage autophagy increased significantly and resulted in a dramatic clearance of macrophages in the Akt inhibitor, mTOR inhibitor or mTOR-siRNA treated groups. The mRNA or protein expression levels of autophagy related gene or protein Beclin 1, LC3-II, Atg5-Atg12 conjugation and typical autophagosome in the macrophages were increased significantly after the Akt and mTOR inhibitors or mTOR gene silencing treatment. In the meanwhile, macrophage RAM-11 staining was significantly reduced as compared to the control group. What's more, the amount of SMC changed little after autophagy was induced in the experimental group than that of the control. SMC which mainly expressed in the cap of the plaque can secrete collagen, thus is valued for its protective role on the plaque rupture.

PI3K has different types and each has a different role in the regulation of autophagy. The class I PI3K signaling inhibits autophagy because it can activate the major downstream molecule of Akt and mTOR [18]. Therefore, PIK3 inhibitor LY294002 could also inhibit Akt or mTOR mRNA expression as shown in our present study. In contrast, class III PI3K signaling can enhance autophagy by induction of beclin 1 [19]. In our experiment, Beclin l overexpressed in group B1, C1 and D1 compared to group E1, which means an induction of autophagy by class III PI3K. We hold a view that LY294002 has more influence on class I PI3K but autophagy is mostly regulated via class III PI3K.

In the process of atherosclerosis, a number of factors mediate mTOR activation and subsequently down-regulate the macrophage autophagy. Recent studies in organ transplant have revealed that immunosuppressant everolimus could cause severe hyperlipidemia and even acute pancreatitis [20]. Everolimus is an mTOR inhibitor commonly used for post-transplant immunosuppression. The pathogenesis of hyperlipidemia associated with mTOR inhibitor may be related to decreased degradation of apolipoprotein B100 [21]. You et al found that vasoactive peptide urotensin II receptor antagonist can cause hyperlipidemia and decrease mTOR and Kyung et al showed that phosphatase and tensin homolog (PTEN) inactivation may have relationship with activation of the PI3K/Akt signaling pathway [22], [23]. Researches focusing on lipid study proved that changes in the intracellular lipid content reduced autophagy both in vitro experiment and high fat diet fed animal, unsaturated fatty acids (FFA) such as oleic acid had a marked stimulatory effect on autophagy while saturated FFA such as palmitic acid suppressed autophagy [24]. The increasing saturated FFA concentration is one of features in atherosclerosis patients. Mechanism for this phenomenon showed that inducing caspase-dependent Beclin 1 cleavage was responsible [25]. Jia et al proved that insulin-like growth factor-1 (IGF-1) could inhibit autophagy in plaque through the Akt pathway [26].

In our vivo study, a rabbit model of vulnerable plaques fed on high-fat diet was obtained by endothelial injury as reported previously [9]. Our recent study had reported that mTOR inhibitor rapamycin had an effect of inhibition of plaque inflammation independent of serum lipid levels [27]. In the present study, we also found that serum lipid profile changed little in the treatment groups. The plaque formed in the rabbit abdominal aorta had a large lipid core, a thin cap and abundant macrophages, which are features of vulnerable plaques that are seen in humans as well. Several results acquired from our experiment supported the conclusion that selective inhibition of Akt/mTOR signaling pathway significantly attenuated atherosclerotic plaque inflammation, burden and vulnerability: at first, the macrophages in the plaques and the inflammatory factors such as MMP9 secreted by macrophage that promoted the destabilization and rupture of atherosclerotic plaques were proved to be decreased; furthermore, the vulnerability index of the atherosclerotic plaque was decreased consistently; and then, at the end of week 16, IVUS measurements of PA and PB% were greatly decreased in the treatment group; and finally, the ratios of rabbits which developed plaque disruption in the treatment groups were significantly lower than the control group, which meant the pathway inhibitors or gene silencing successfully prevented vulnerable plaques from rupture.

Cytokines have an important role in regulating autophagy via different pathways. It has been known that IFN-γ had an impact on activating macrophages in a model when cells were infected with mycobacteria, leading to maturation of autophagosome [28]. Experiment showed that IFN-γ induced autophagy could be abrogated by the TNF blockers adalimumab and infliximab, which suggested that it might be dependent on TNF-α [29]. Jia et al reported that in human atherosclerotic vascular smooth cells, TNF-α increases expression of the autophagy genes LC3 and Beclin 1 depending on the Jun kinase (JNK) pathway, as well as the inhibition of Akt activation [26]. In our experiment, we also found that expression level of IFN-γ was decreased in the PI3K inhibitor LY294002 group while increased after treated with triciribine, rapamycin or mTOR-siRNA. IL-10 was chosen into the present study because it could inhibit various types of inflammatory cells [30]. IL-10 inhibited starvation-induced autophagy; most likely it activated class I PI3K signaling and thus inhibited the Akt pathway ultimately [31].

Our study contains several limitations. First, the animal number in each group is small especially after pharmacological triggering and thus a larger sample of animals and other types of animals, such as apoE-/- mice, should apply to confirm our results. Second, although 8 week triciribine and rapamycin treatment effectively stabilized vulnerable plaques, the side-effect of long term use of these drugs and possible changes of plaque vulnerability was not involved in this study, thus, further studies are required to work out the necessity and safety of long-term administration of triciribine and rapamycin.

In conclusion, selectively inhibition of Akt/mTOR pathway can inhibit the atherosclerosis progression and enhance the stability of atherosclerotic plaques by activation of macrophage autophagy.

## Materials and Methods

### In vitro experiments

#### Cell culture

The rabbit peritoneal macrophages were isolated 4 days after injecting thioglycollate broth medium into the peritoneal cavity of New Zealand rabbit. The cells were seeded in RPMI 1640 medium supplemented with 100 U/ml penicillin, 100 µg/ml streptomycin, and 10% fetal bovine serum and incubated in 5% CO_2_ at 37°C. Cells were then treated with PI3K inhibitor LY294002 (10 µmol/L, group A1) [32], Akt inhibitor triciribine (20 µmol/L, group B1) [33], mTOR inhibitor rapamycin (10 ng/mL, group C1) [34], mTOR-siRNA (30 nmol/L, group D1) [35] and control group (E1) for 48 hours, respectively. For mTOR gene silencing experiments, cells were transfected with 30 nmol/l short interfering (si) RNA specific to mTOR (Guangzhou Ribobio Co., Lid) using lipofectamine 2000 transfection reagent (life technologies Cor.) according to the instructions of the manufacturer. For all data shown, individual experiments were repeated three times.

#### Immunofluorescence staining

Cells were seeded into slides and incubated with LY294002, triciribine, rapamycin, mTOR-siRNA for 48 hours, respectively and then were fixed with 4% paraformaldehyde for 30 minutes at 4°C. After washing with PBS, cells were punched with 1‰ Triton and then incubated with mouse anti-rabbit LC3-II antibody at 4°C overnight. The slides were washed with PBS and then incubated with mouse anti-rabbit IgG-FITC-conjugated antibody for 2 h at room temperature. Cell nuclei were counterstained by exposure to DAPI (1 µg/mL). After a final wash, the slides were mounted and analyzed under a laer scanning confocal microscope.

#### Enzyme linked immunosorbent assay (ELISA)

Cells supernatant was collected after 48 hours of co-incubation with drugs or mTOR-siRNA from the five groups and IFN-γ, IL-10 were measured by ELISA kits (eBioscicence, Inc.), respectively. Operations were strictly performed for three times following the instructions, and the values were corrected by the contents of the total sample protein.

#### Transmission electron microscope

Cultured macrophages or atherosclerotic segments were fixed in 0.1 mol/L sodium cacodylate-buffered (pH 7.4) 2.5% glutaraldehyde solution for 2 h, then rinsed (3×10 minutes) in 0.1 mol/L sodium cacodylate-buffered (pH 7.4) 7.5% saccharose and postfixed in 1% OsO_4_ solution for 1 hour. After dehydration in an ethanol gradient (70% ethanol for 20 min, 96% ethanol for 20 min, 100% ethanol for 2×20 min), samples were embedded in Durcupan ACM and then were stained with uranylacetate and lead citrate. Sections were examined under a Japan JEM-1200 microscope at 80 kV.

#### Quantitative RT-PCR

The mRNA expression levels of Akt, mTOR and Beclin 1 genes were performed using reverse-transcription polymerase chain reaction (RT-PCR). The mRNA was extracted from macrophages with a RNA isolation kit (Thermo Scientific Molecular Biology). 2 µg of total RNA was reverse transcribed into cDNA using SYBR Green. PCR was performed at the conditions of 95°C for 2 min, followed by 35 cycles of 95°C for 15 s, 55°C for 15 s, and 68°C for 20 s. The transcript number of GAPDH was quantified as an internal control. Three replicates were run for each sample in a 96-well and 200 ng RNA were used for each reaction. Results were analyzed using the ΔΔCt method.

#### Western blot

Total protein was resolved on SDS-PAGE and transferred onto a nitrocellulose membrane. After blocking in 5% non-fat milk (in Tris-Buffered-Saline with Tween, TBST) for 2 hours, membranes were incubated with primary antibodies: mouse anti-rabbit mTOR monoclonal antibody (Cell signaling, USA), P-mTOR monoclonal antibody (Cell signaling, USA), Akt monoclonal antibody (Cell signaling, USA), P-Akt monoclonal antibody (Sigma, USA), LC3-II monoclonal antibody (Cell signaling, USA), Atg5-Atg12 conjugation monoclonal antibody (Merck Millipore Cor., Germany) and β-actin monoclonal antibody (Zhongshan Cro. China) overnight at 4°C. Membranes were washed with TBST for 3 times following by incubation with corresponding secondary antibodies. Expression of individual proteins was normalized to that of β-actin. Western blot was repeated at least three times.

### In vivo experiments

#### Animal model and ethics statement

In vitro study, we found that LY294002 had a role of inhibition of macrophage autophagy and had no effect on its subtype PI3K-I, so we abandoned the use of LY294002 and divided only four groups to verify our hypothesis in vivo. Forty New Zealand rabbits weighing from 2.0 to 3.0 kg were randomly divided into four groups (n = 10 each). Balloon-induced aortic wall injury was performed with a 4-Fr balloon catheter (3.5×15 mm^2^) introduced through the right femoral artery to the thoracic aorta after rabbits had been anesthetized with an intravenous injection of pentobarbital sodium (30 mg/kg). The balloon was inflated with saline to obtain 8 atm, and the catheter was retracted down to the iliofemoral artery. This process was repeated three times in each rabbit to ensure denudation of the endothelium of the abdominal aorta. Then rabbits were fed on a diet of 1% cholesterol for 16 weeks. From the end of week 8 to the end of week 16, rabbits in group A2 were given a daily oral dose of triciribine (1.0 mg·kg^−1^·d^−1^) [34], rabbits in group B2 received a daily oral dose of rapamycin (0.5 mg·kg^−1^·d^−1^) [36], rabbits in group D2 received a daily oral dose of PBS (1.0 ml·kg^−1^·d^−1^) as the control group. These drugs were dissolved in water and given by oral gavage every day. For rabbits in C2 group, siRNA dissolved in RNAse-free and endotoxin-free water at 3 µg/µl and then diluted in PBS (500 µg/2 ml per rabbit) were injected intraperitoneally (i.p.) once a week for 8 weeks [37]. At the end of week 16, plaque rupture was induced in all rabbits by pharmacological triggers using the method as described previously [9]. In brief, 0.15 mg·kg^−1^ of Chinese Russell's viper venom was injected intraperitoneally, followed 30 min later by an intravenous injection of 0.02 mg·kg^−1^ histamine (Sigma, USA). Twenty-four hours after pharmacological triggering, rabbits were sacrificed for pathological studies. All animal care and experimental protocols complied with the Animal Management Rules of the Ministry of Health of the People's Republic of China (document No 55, 2001) and were approved by the Animal Care Committee of Shandong University.

#### Biochemical assays

At the beginning of the experiment and the end of week 16, blood samples were collected from all rabbits before euthanasia. Serum levels of total cholesterol (TC), triglyceride (TG), high-density lipoprotein cholesterol (HDL-C), and low-density lipoprotein cholesterol (LDL-C) were measured by enzymatic assays.

#### IVUS studies

Rabbits received an IVUS studies before and after pharmacological triggering by use of a 3.2 F catheter which contained a single rotating element transducer of 40 MHz connected to an IVUS system (iLab, Boston Scientific Cor., USA). The catheter was pulled back from the aortic arch to the abdominal aorta by a motorized withdrawal device at a constant speed of 0.5 mm·s^−1^. The following parameters were measured from the abdominal aortic cross-sectional images: external elastic membrane area (EEMA), lumen area (LA), plaque area (PA = EEMA−LA) and plaque burden (PB% =  PA/EEMA ×100%).

#### Immunohistochemistry

Rabbits were sacrificed by intravenous overdose of pentobarbital. The occurrence of plaque rupture and thrombosis of abdominal aorta was observed. Tissue samples (2 cm long) were cut from the abdominal aorta and fixed in 4% form-aldehyde. Tissue samples embedded in paraffin were reacted with mouse anti-rabbit RAM-11 mono-clonal antibody (Dako, USA), mouse anti-rabbit α-smooth-muscle-cell (SMC) actin monoclonal antibody (Sigma, USA), mouse anti-rabbit MMP9 monoclonal antibody (Chemicon International Inc., USA), mouse anti-rabbit mTOR monoclonal antibody (Cell, signaling, USA), mouse anti-rabbit Atg5-Atg12 conjugation monoclonal antibody (Merck Millipore Cor., Germany). The sections were incubated with a goat anti-mouse peroxidase-labeled anti-body (ZSGB-BIO) as secondary antibody for 15 min. A computer-assisted morphometric analysis system (Image-Pro Plus 5.0, Media Cybernetics, USA) were used for histopathological slides analysis. The positive staining of α-actin (SMCs), RAM-11 (macrophages), mTOR and Atg5-Atg12 conjugation in plaque region was counted in five images of every slice under high-power fields (×400) and three individuals were chosen to represent one group.

#### Sirius red staining

Specimen sections were deparaffinized, rehydrated and incubated with 0.1% Sirius red (Bio-technology, USA) in saturated picric acid for 60 minutes. After incubation in 1% acetic acid for 30 minutes and rinsing, slides were counterstained in hematoxylin, differentiated in acid alcohol solution, rehydrated and mounted. Slides were visualized under both bright-field and polarized light microscope, and pictures were taken with identical exposure settings for all sections. The content of collagen type I and III were identified by birefringence under polarized light.

#### Oil red O staining

Sections from each group specimen were incubated in 60% isopropanol for 2 minutes and then in oil red O (Bio-technology, USA) solution for 20 minutes and rinsed in H2O, after that the sections were counterstained with hematoxylin.

### Statistical analysis

Values were expressed as mean ± SD for each condition and analyzed with SPSS software 17.0. Comparison of continuous variables among multiple groups was performed by analysis of variance with one-way ANOVA and post hoc comparisons were made using Limited Slip Differential (LSD) at homogeneity of variance or Dunnett's T3 at heterogeneity of variance. Two-tailed *P*<0.05 was considered statistically significant.
